# Supplementary material for: Long-term survival of female versus male patients after coronary artery bypass grafting
Source: PLoS One. 2022 Sep 23;17(9):e0275035. doi: 10.1371/journal.pone.0275035 (PMC9506631; doi:10.1371/journal.pone.0275035)
Supplement: S1 Table — (DOCX) [file pone.0275035.s001.docx]

**S1 Table.** International Classification of Diseases, 9th Edition, Clinical Modification codes of the conditions defined as exclusion criteria

| **Exclusion criteria** | **International Classification of Diseases, 9th Edition,**  **Clinical Modification codes** |
| --- | --- |
| **Previous cardiac surgery** | V433, V4581 |
|  |  |
| **Multiple valve surgery**  **Thoracic aorta surgery** | 3510, 3511, 3512, 3513, 3514, 3520, 3521, 3522, 3523, 3524, 3525, 3526, 3527, and 3528 |
|  |  |
|  | 3804, 3814, 3834, 3844, 3845, and 3864 |
|  |  |
| **Mechanical complications** | 41410, 4295, 4296, 42971, and 42979 |
