# Supplementary material for: Long-term survival of female versus male patients after coronary artery bypass grafting
Source: PLoS One. 2022 Sep 23;17(9):e0275035. doi: 10.1371/journal.pone.0275035 (PMC9506631; doi:10.1371/journal.pone.0275035)
Supplement: S2 Table — (DOCX) [file pone.0275035.s002.docx]

**S2 Table.** International Classification of Diseases, 9th Edition, Clinical Modification codes of the conditions defined as covariates.

| **Covariate** | **International Classification of Diseases, 9th Edition,**  **Clinical Modification codes** |
| --- | --- |
| **Hypertension** | 401, 4011, 4019, 402, 4020, 4021, 4029, 40290, 40291, 403, 4030, 40300, 40301, 4031, 40310, 40311, 4039, 40390, 40391, 404, 4040, 40400, 40401, 40402, 40403, 4041, 40410, 40411, 40412, 40413, 4049, 40490, 40491, 40492, 40493, 405, 4050, 4050, 40509, 4051, 40511, 40519, 4059, 40591, 40599, and 4372 |
| **Diabetes mellitus**  Non-insulin treated  Insulin treated | 249, 2490, 24900, 24901, 2491, 24910, 24911, 2492, 24920, 24921, 2493, 24930, 24931, 2494, 24940, 24941, 2495, 24950, 24951, 2496, 24960, 24961, 2497, 24970, 24971, 2498, 24980, 24981, 2499, 24990, 24991, 250, 2500, 25000, 25001, 25002, 25003, 2501, 25010, 25011, 25012, 25013, 2502, 25020, 25021, 25022, 25023, 2503, 25030, 25031, 25032, 25033, 2504, 25040, 25041, 25042, 25043, 2505, 25050, 25051, 25052, 25053, 2506, 25060, 25061, 25062, 25063, 2507, 25070, 25071, 25072, 25073, 2508, 25080, 25081, 25082, 25083, 2509, 25090, 25091, 25092, and 25093  V5867 |
| **Hyperlipidemia** | 2720, 2721, 2722, 2723, 2724, 2725, 2726, 2727, 2728, and 2729 |
|  |  |
| **Obesity** | 27800, 278012, 27802, and 27803 |
|  |  |
| **Smoking status**  Previous smoker  Current smoker | V1582  3051 |
|  |  |
| **Cerebrovascular disease** | 3623, 36230, 36231, 36232, 36233, 36234, 36235, 36236, 36237, 3466, 34660, 34661, 34662, 34663, 430, 431, 432, 4320, 4321, 4329, 433, 4330, 43300, 43301, 4331, 43310, 43311, 4332, 43320, 43321, 4333, 43330, 43331, 4338, 43380, 43381, 4339, 43390, 43391, 434, 4340, 43400, 43401, 4341, 43410, 43411, 4349, 43490, 43491, 435, 4350, 4351, 4352, 4353, 4358, 4359, 436, 437, 4370, 4371, 4372, 4373, 4374, 4375, 4376, 4377, 78043, 99702, 4378, 4379, 438, 4380, 4381, 43810, 43811, 43812, 43813, 43814, 43819, 4382, 43820, 43821, 43822, 4383, 43830, 43831, 43832, 4384, 43840, 43841, 43842, 4385, 43850, 43851, 43852, 4386, 4387, 4388, 43881, 43882, 43883, 43884, 43885, 43889, and 4389 |
|  |  |
| **Congestive heart disease** | 39891, 428, 4280, 4281, 4282, 42820, 42821, 42822, 42823, 4283, 42830, 42831, 42832, 42833, 4284, 42840, 42841, 42842, 42843, 4289, 429, 4290, 4291, 4292, 4293, 4294, 4295, 4296, 4297, 42971, 42979, 4298, 42981, 42982, 42983, 42989, and 4299 |
|  |  |
| **Peripheral vascular disease** | 4400, 4401, 4402, 44020, 44021, 44022, 44023, 44024, 44029, 4403, 44030, 44031, 44032, 4404, 4408, 4409, 4471, 9961, 99662, 99674, and V434 |
|  |  |
| **Chronic obstructive pulmonary disease** | 491, 4910, 4911, 4912, 49120, 49121, 49122, 4918, 4919, 492, 4920, 4928, 493, 4930, 49300, 49301, 49302, 4931, 49310, 49311, 49312, 4932, 49320, 49321, 49322, 4938, 49380, 49381, 49382, 4939, 49390, 49391, 49392, 494, 4940, 4941, and 496 |
|  |  |
| **Chronic kidney disease**  Non-dialysis dependent  Dialysis dependent | 403, 4030, 40300, 40301, 4031, 40310, 40311, 4039, 40390, 40391, 404, 4040, 40400, 40401, 40402, 40403, 4041, 40410, 40411, 40412, 40413, 4049, 40490, 40491, 40492, 40493, 582, 5820, 5821, 5822, 5824, 5828, 58281, 58289, 5829, 583, 5830, 5831, 5832, 5834, 5836, 5837, 5838, 58381, 58389, 5839, 585, 5851, 5852, 5853, 5854, 5855, 5859, 586, 587, 588, 5880, 5881, 5888, 58881, 58888, 5889, V420, and V56  V560, V561, V562, V563, V5631, V5632, V568, and 5856 |
|  |  |
| **Liver disease** | 070, 0700, 0701, 0702, 07020, 07021, 07022, 07023, 0703, 07030, 07031, 07032, 07033, 0704, 07041, 07042, 07043, 07044, 07049, 0705, 07051, 07052, 07053, 07054, 07059, 0706, 0707, 07070, 07071, 0709, 456, 4560, 4561, 4562, 45620, 45621, 4563, 4564, 4565, 4566, 4568, 570, 571, 5710, 5711, 5712, 5713, 5714, 57140, 57141, 57142, 57149, 5715, 5716, 5718, 5719, 572, 5720, 5721, 5722, 5723, 5724, 5728, 573, 5730, 5731, 5732, 5733, 5734, 5735, 5738, 5738, 7824, 7891, 7895, 78951, 78959, 7904, 7905, 7948, and V427 |
|  |  |
| **Anemia** | 280, 2800, 2801, 2808, 2809, 281, 2810, 2811, 2812, 2813, 2814, 2818, 2819, 282, 2820, 2821, 2822, 2823, 2824, 28240, 28241, 28242, 28243, 28244, 28245, 28246, 28247, 28249, 2825, 2826, 28260, 28260, 28261, 28262, 28263, 28264, 28268, 28269, 2827, 2828, 2829, 283, 2830, 2831, 28310, 28311, 28319, 2832, 2839, 284, 2840, 28401, 28409, 2848, 28481, 28489, 2849, 285, 2850, 2852, 28521, 28522, 28529, 2853, 2858, and 2859 |
|  |  |
| **Coagulation disorders** | 286, 2861, 2862, 2863, 2864, 2865, 28652, 28653, 28659, 2866, 2867, 2869, 287, 2870, 2871, 2872, 2873, 28730, 28731, 28732, 28733, 28739, 2874, 2875, 2878, and 2879 |
|  |  |
| **Cancer**  Oropharyngeal cancer  Gastrointestinal cancer  Respiratory tract cancers  Bone and connective tissue cancers  Genitourinary cancers  Lymphoid cancers  Hematologic cancers  Other cancers | 140, 1400, 1401, 1403, 1404, 1405, 1406, 1408, 1409, 141, 1410, 1411, 1412, 1413, 1414, 1415, 1416, 1418, 1419, 142, 1420, 1421, 1422, 1428, 1429, 143, 1430, 1431, 1438, 1439, 144, 1440, 1441, 1448, 1449, 145, 1450, 1451, 1452, 1453, 1454, 1455, 1456, 1458, 1459, 146, 1460, 1461, 1462, 1463, 1464, 1465, 1466, 1467, 1468, 1469, 147, 1470, 1471, 1472, 1473, 1478, 1479, 148, 1480, 1481, 1482, 1483, 1488, 1489, 149, 1490, 1491, 1498, and 1499  150, 1500, 1501, 1502, 1503, 1504, 1505, 1506, 1508, 1509, 1510, 151, 1511, 15012, 1513, 1514, 1515, 1516, 1518, 1519, 152, 1520, 1521, 1522, 1523, 1528, 1529, 153, 1530, 1531, 1532, 1533, 1534, 1535, 1536, 1537, 1538, 1539, 154, 1540, 1541, 1542, 1543, 1548, 155, 1550, 1551, 1552, 156, 1560, 1561, 1562, 1568, 1569, 157, 1570, 1571, 1572, 1573, 1574, 1578, 1579, 158, 1580, 1588, 1589, 159, 1590, 1591, 1598, 1599  160, 1600, 1601, 1602, 1603, 1604, 1605, 1608, 1609, 161, 1610, 1611, 1612, 1613, 1618, 1619, 162, 1620, 1622, 1623, 1624, 1625, 1628, 1629, 163, 1630, 1631, 1638, 1639, 164, 1640, 1461, 1462, 1463, 1468, 1649, 165, 1650, 1658, and 1659  170, 1700, 1701, 1702, 1703, 1704, 1705, 1706, 1707, 1708, 1709, 171, 1710, 1712, 1713, 1714, 1715, 1716, 1717, 1718, 1719, 172, 1720, 1721, 1722, 1723, 1724, 1725, 1726, 1727, 1728, 1729, 173, 1730, 17300, 17301, 17302, 17309, 1731, 17310, 17311, 17312, 17319, 1732, 17320, 17321, 17322, 17329, 1733, 17330, 17331, 17332, 17339, 1734, 17340, 17341, 17342, 17349, 1735, 17350, 17351, 17352, 17359, 1736, 17360, 17361, 17362, 17369, 1737, 17370, 17371, 17372, 17379, 1738, 17380, 17381, 17382, 17389, 1739, 17390, 17391, 17392, 17399, 174, 1740, 1741, 1742, 1743, 1744, 1745, 1746, 1748, 1749, 1752, 1750, 1759, 176, 17602, 1761, 1762, 1763, 1764, 1765, 1768, and 1769  179, 180, 1800, 1801, 1808, 1809, 181, 182, 1820, 1821, 1828, 183, 1830, 1832, 1833, 1834, 1835, 1838, 1839, 184, 1840, 1841, 1842, 1843, 1844, 1848, 1849, 185, 186, 1860, 1869, 187, 1871, 1872, 1873, 1874, 1875, 1876, 1877, 1878, 1879, 188, 1880, 1881, 1882, 1883, 1884, 1885, 1886, 1887, 1888, 1889, 189, 1890, 1891, 1892, 1893, 1894, 1898, and 1899  200, 2000, 20000, 20001, 20002, 20003, 20004, 20005, 20006, 20007, 20008, 2001, 20010, 20011, 20012, 20013, 20014, 20015, 20016, 20017, 20018, 2002, 20020, 20021, 20022, 20023, 20024, 20025, 20026, 20027, 20028, 2003, 20030, 20031, 20032, 20033, 20034, 20035, 20036, 20037, 20038, 2004, 20040, 20041, 20042, 20043, 20044, 20045, 20046, 20047, 20048, 2005, 20050, 20051, 20052, 20053, 20054, 20055, 20056, 20057, 20058, 2006, 20060, 20061, 20062, 20063, 20064, 20065, 20066, 20067, 20068, 2007, 20070, 20071, 20072, 20073, 20074, 20075, 20076, 20076, 20077, 20078, 2008, 20080, 20081, 20082, 20083, 20084, 20085, 20086, 20087, 20088, 201, 2010, 20100, 20101, 20102, 20103, 20104, 20105, 201062, 20107, 20108, 2011, 20110, 20111, 20112, 20113, 20114, 20115, 20116, 20117, 20118, 2012, 20120, 20121, 20122, 20123, 20124, 20125, 20126, 20127, 20128, 2014, 20140, 20141, 201422, 20143, 20144, 20145, 20146, 20147, 20148, 2015, 20150, 20151, 20152, 20153, 20154, 20155, 20156, 20157, 20158, 2016, 20160, 20161, 20162, 20163, 20164, 20165, 20166, 20167, 20168, 2017, 20170, 20171, 20172, 20173, 20174, 20175, 20176, 20177, 20178, 2019, 201902, 20191, 20192, 20193, 20194, 20195, 20196, 20197, 20198, 202, 2020, 20200, 20201, 20202, 20203, 20204, 20205, 20206, 20207, 20208, 2021, 20210, 20211, 20212, 20213, 20214, 20215, 20216, 20217, 20218, 2022, 20220, 20221, 20222, 20223, 20224, 20225, 20226, 20227, 20228, 2023, 20230, 20231, 20232, 20233, 20234, 20235, 20236, 202372, 20238, 2024, 20240, 20241, 20242, 20243, 20244, 20245, 20246, 20247, 20248, 2025, 20250, 20251, 20252, 20253, 20254, 20255, 20256, 20257, 20258, 2026, 20260, 20261, 20262, 20263, 20264, 20265, 20266, 20267, 20268, 2027, 20270, 20271, 20272, 20273, 20274, 20275, 20276, 20277, 20278, 2028, 20280, 20281, 20282, 20283, 20284, 20285, 20286, 20287, 20288, 2029, 20290, 20291, 20292, 20293, 20294, 20295, 20296, 20297, 20298, 203, 20302, 20300, 20301, 20302, 2031, 20310, 20311, 20312, 2038, 203802, 20381, and 20382  204, 2040, 20400, 20401, 20402, 2041, 20410, 20411, 20412, 2042, 204202, 20421, 20422, 2048, 20480, 20481, 20482, 2049, 20490, 20491, 20492, 205, 2050, 20500, 20501, 20502, 2051, 20510, 20511, 20512, 2052, 20520, 20521, 20522, 2053, 20530, 20531, 20532, 2058, 20580, 20581, 20582, 2059, 20590, 20591, 20592, 206, 2060, 20600, 20601, 20602, 2061, 20610, 20611, 20612, 2062, 20620, 20621, 20622, 2068, 20680, 20681, 20682, 2069, 20690, 20691, 20692, 207, 2070, 20700, 20701, 20702, 2071, 20710, 20711, 20712, 2072, 20720, 20721, 20722, 2078, 20780, 20781, 20782, 208, 2080, 20800, 20801, 20802, 2081, 20810, 20811, 20812, 2082, 20820, 20821, 20822, 2088, 20880, 20881, 20882, 2089, 20890, 20891, 20892, 209, 2090, 20900, 20901, 20902, 20903, 2091, 20910, 20911, 20912, 20913, 20914, 20915, 20916, 20917, 20922, 20920, 20921, 20922, 20923, 20924, 20925, 20926, 20927, 20929, 2093, 20930, 20931, 20932, 20933, 20934, 20935, 20936, 2094, 20940, 20941, 20942, 20943, 2095, 20950, 20951, 20952, 20953, 20954, 20955, 20956, 20957, 2096, 20960, 20961, 20962, 20963, 20964, 20965, 20966, 20967, 20969, 2097, 209702, 20971, 20972, 20973, 20974, 20975, and 20979  190, 1900, 1901, 1902, 1903, 1904, 1905, 1906, 1907, 1908, 1909, 191, 1910, 1911, 1912, 1913, 1914, 1915, 1916, 1917, 1918, 1919, 192, 1920, 1921, 1922, 1923, 1928, 1929, 193, 194, 1940, 1941, 1943, 1944, 1945, 1946, 1948, 1952, 1950, 1951, 1952, 1953, 1954, 1955, 1958, 196, 1960, 1961, 1962, 1963, 1965, 1966, 1968, 1969, 197, 1970, 1971, 1972, 1973, 1974, 1975, 1976, 1977, 1978, 1982, 1980, 1981, 1982, 1983, 1984, 1985, 1986, 1987, 1988, 19881, 19882, 19889, 199, 1990, 1991, and 1992 |
