# Supplementary material for: Long-term survival of female versus male patients after coronary artery bypass grafting
Source: PLoS One. 2022 Sep 23;17(9):e0275035. doi: 10.1371/journal.pone.0275035 (PMC9506631; doi:10.1371/journal.pone.0275035)
Supplement: S3 Table — (DOCX) [file pone.0275035.s003.docx]

**S3 Table.** International Classification of Diseases, 9th Edition, Clinical Modification codes of the conditions defined as outcomes

| **Outcome** | **International Classification of Diseases, 9th Edition,**  **Clinical Modification codes** |
| --- | --- |
| **Perioperative stroke** | 99701, and 99702 |
| **Cardiac complications**  POAF  Permanent pacemaker  IABP  Cardiac arrest | 42731, and 42732 (excluding V5681)  3780, 3781, 3782, and 3783  3761  4275 |
|  |  |
| **Pulmonary complications**  Prolonged ventilation  Reintubation  Tracheotomy  **Acute kidney injury**  **Hemorrhage** | 9670, 9671, and 9672  9604  311 |
|  |  |
|  | 5845, 5846, 5847, 5848, 5849, and 9975 |
|  |  |
|  | 9981, 99811, and 99812 |
|  |  |
| **RBC transfusion** | 9904 |
|  |  |
| **Wound complications** | 99830, 99831, 99832, and 99859 |
