# Supplementary material for: Long-term survival of female versus male patients after coronary artery bypass grafting
Source: PLoS One. 2022 Sep 23;17(9):e0275035. doi: 10.1371/journal.pone.0275035 (PMC9506631; doi:10.1371/journal.pone.0275035)
Supplement: Attachment — Submitted filename: Response to Reviewers.docx [file pone.0275035.s005.docx]

**Armando José Pimenta de Abreu**

Cardiothoracic Surgery Department

Centro Hospitalar Universitário São João

Alameda Hernâni Monteiro

4200-319 Porto, Portugal

00 351 912 263 994

armandopabreu@gmail.com

2 August 2022

**Alessandro Leone, MD**
Academic Editor
PLOS ONE

**Reply to review**

**Journal Requirements:**

**1. Please ensure that your manuscript meets PLOS ONE's style requirements, including those for file naming.**

**Answer**: We have devoted the best of our efforts to meet PLOS ONE’s style requirements. Likewise, files have been renamed as instructed.

**2. Please provide additional details regarding participant consent. In the ethics statement in the Methods and online submission information, please ensure that you have specified what type you obtained (for instance, written or verbal, and if verbal, how it was documented and witnessed). If your study included minors, state whether you obtained consent from parents or guardians. If the need for consent was waived by the ethics committee, please include this information.**

**Answer**: The need for consent was waived by the ethics committee. We have updated the Ethics subsection of our manuscript to include this information.

**3. We note that you have indicated that data from this study are available upon request. PLOS only allows data to be available upon request if there are legal or ethical restrictions on sharing data publicly.**

**Answer**: As requested, we have uploaded the minimal anonymized data set necessary to replicate our study findings as a Supporting Information file.

**4. Please amend the manuscript submission data (via Edit Submission) to include author Alberto Freitas.**

**Answer**: The inclusion of Professor Alberto Freitas as author of the present paper was an unfortunate lapse. Therefore, his name has been removed from the first page of the manuscript.

**5. Please include captions for your Supporting Information files at the end of your manuscript, and update any in-text citations to match accordingly.**

**Answer**: Supporting Information captions have been included at the end of the manuscript in a section titled “Supporting information”.

**6. Please review your reference list to ensure that it is complete and correct. If you have cited papers that have been retracted, please include the rationale for doing so in the manuscript text, or remove these references and replace them with relevant current references. Any changes to the reference list should be mentioned in the rebuttal letter that accompanies your revised manuscript. If you need to cite a retracted article, indicate the article’s retracted status in the References list and also include a citation and full reference for the retraction notice.**

**Answer**: We confirm that our reference list is complete and correct. To the best of our knowledge, none of the cited papers has been retracted.

**Reviewers' comments:**

**Reviewer's Responses to Questions**

**Comments to the Author**

**1. Is the manuscript technically sound, and do the data support the conclusions?

The manuscript must describe a technically sound piece of scientific research with data that supports the conclusions. Experiments must have been conducted rigorously, with appropriate controls, replication, and sample sizes. The conclusions must be drawn appropriately based on the data presented.**

**Reviewer #1: Yes**

**Reviewer #2: Yes**

**2. Has the statistical analysis been performed appropriately and rigorously?**

**Reviewer #1: Yes**

**Reviewer #2: Yes**

**3. Have the authors made all data underlying the findings in their manuscript fully available?

The** [**PLOS Data policy**](http://www.plosone.org/static/policies.action#sharing) **requires authors to make all data underlying the findings described in their manuscript fully available without restriction, with rare exception (please refer to the Data Availability Statement in the manuscript PDF file). The data should be provided as part of the manuscript or its supporting information, or deposited to a public repository. For example, in addition to summary statistics, the data points behind means, medians and variance measures should be available. If there are restrictions on publicly sharing data — e.g., participant privacy or use of data from a third party—those must be specified.**

**Reviewer #1: Yes**

**Reviewer #2: Yes**

**4. Is the manuscript presented in an intelligible fashion and written in standard English?

PLOS ONE does not copyedit accepted manuscripts, so the language in submitted articles must be clear, correct, and unambiguous. Any typographical or grammatical errors should be corrected at revision, so please note any specific errors here.**

**Reviewer #1: Yes**

**Reviewer #2: Yes**

**5. Review Comments to the Author**

**Please use the space provided to explain your answers to the questions above. You may also include additional comments for the author, including concerns about dual publication, research ethics, or publication ethics. (Please upload your review as an attachment if it exceeds 20,000 characters)**

**Reviewer #1: This manuscript represents a well written population level analysis investigating female sex and long-term all-cause mortality with revascularization. The data is well described and relatively complete with findings of no prognostic implications with female sex 10 years-procedure. Consistent with national trends an almost 4:1 ratio of men.**

**Answer**: We are most obliged by your kind remarks.

**Reviewer #2: Dear the authors of the manuscript entitled “Long-term survival of female versus male patients after coronary artery bypass grafting”**

**I want to thank you for your efforts in writing this retrospective study which compares the long-term survival between males and females who underwent coronary artery bypass grafting. This study included good number of patients in both cohorts and had excellent long term follow up period. It is obvious that in the non-adjusted cohorts, that female patients are at having comorbidity risks and received lesser chances of arterial revascularization as well as completeness of revascularization, and that is typical what we believe in and see in our daily practice. Contrary to what we know, and I think that is the beauty of this study, once we match these cohorts, survival was comparable between the 2 cohorts, and that is a fact which is good to provide. Since our decisions in the choice of revascularization strategy are sometimes governed by the gender of the patients, I think this study is good to add to the literature.
My points here to the authors:**

1. **Did you have any data regarding the syntax score for assessment of the quality of the coronary arteries in both cohorts? I know it is sometimes hard to find this piece of information for all the patients, however if there is available data about this, I guess it will add much to this study.**

**Answer**: We are thankful for your insightful analysis. Unfortunately, the Syntax Score is not routinely calculated in our center. Therefore, this important information is unavailable for analysis.

2. **Any idea about the echo assessment of the left ventricular function for both cohorts?**

**Answer**: One of the fundamental problems when using administrative datasets for clinical profiling in cardiac surgery is that certain fundamental variables in all risk score calculations are unavailable. One of such variables is precisely left ventricular function. However, based on the available literature where this item is reported, we should not expect substantial differences in LV function between both groups.

3. **Do both cohorts had certain type of medication protocol that was identical between the 2 cohorts (Double vs single antiplatelet therapy)?**

**Answer**: The antiplatelet therapy protocol in use in our department does not make gender-based distinctions. We use single antiplatelet therapy in stable coronary artery disease cases and we use DAPT in all OPCAB cases or CABG following an acute coronary syndrome. Both these variables (clinical presentation and use of CPB) were well balanced at baseline. Therefore, we should not expect differences in antiplatelet therapy between the two groups.

**Again, thank you for your efforts in writing this manuscript.**

**6. PLOS authors have the option to publish the peer review history of their article (**[**what does this mean?**](https://journals.plos.org/plosone/s/editorial-and-peer-review-process#loc-peer-review-history)**). If published, this will include your full peer review and any attached files.


If you choose “no”, your identity will remain anonymous but your review may still be made public.


Do you want your identity to be public for this peer review? For information about this choice, including consent withdrawal, please see our** [**Privacy Policy**](https://www.plos.org/privacy-policy)**.**

**Reviewer #1: No**

**Reviewer #2: Yes: Salah Eldien Altarabsheh**

Best regards,

Armando Abreu

Department of Cardiothoracic Surgery, São João University Hospital Center, Porto

Department of Physiology and Surgery, Faculty of Medicine, University of Porto
